# Coherent control schemes for the photoionization of neon and helium in the Extreme Ultraviolet spectral region

**DOI:** 10.1038/s41598-018-25833-7

**Published:** 2018-05-17

**Authors:** Luca Giannessi, Enrico Allaria, Kevin C. Prince, Carlo Callegari, Giuseppe Sansone, Kiyoshi Ueda, Toru Morishita, Chien Nan Liu, Alexei N. Grum-Grzhimailo, Elena V. Gryzlova, Nicolas Douguet, Klaus Bartschat

**Affiliations:** 1 Elettra-Sincrotrone Trieste, 34149 Basovizza, Trieste Italy; 2 ENEA C.R. Frascati, 00044 Frascati, Italy; 3 Dipartimento di Fisica, CNR-IFN, Politecnico di Milano, 20133 Milan, Italy; 4 Physikalisches Institut der Albert-Ludwigs-Universität Freiburg, 79104 Freiburg, Germany; 5 Institute of Multidisciplinary Research for Advanced Materials, Tohoku University, Sendai, 980-8577 Japan; 6 Institute for Advanced Science, The University of Electro-communications, 1-5-1 Chofu-ga-oka, Chofu-shi, Tokyo 182-8585 Japan; 7 Department of Physics, Fu-Jen Catholic University, Taipei, 24205 Taiwan; 8 Skobeltsyn Institute of Nuclear Physics, Lomonosov Moscow State University, Moscow, 119991 Russia; 9 Department of Physics and Astronomy, Drake University, Des Moines, Iowa 50311 USA; 10 Department of Physics, University of Central Florida, Orlando, Florida 32816 USA

## Abstract

The seeded Free-Electron Laser (FEL) FERMI is the first source of short-wavelength light possessing the full coherence of optical lasers, together with the extreme power available from FELs. FERMI provides longitudinally coherent radiation in the Extreme Ultraviolet and soft x-ray spectral regions, and therefore opens up wide new fields of investigation in physics. We first propose experiments exploiting this property to provide coherent control of the photoionization of neon and helium, carry out numerical calculations to find optimum experimental parameters, and then describe how these experiments may be realized. The approach uses bichromatic illumination of a target and measurement of the products of the interaction, analogous to previous Brumer-Shapiro-type experiments in the optical spectral range. We describe operational schemes for the FERMI FEL, and simulate the conditions necessary to produce light at the fundamental and second or third harmonic frequencies, and to control the phase with respect to the fundamental. We conclude that a quantitative description of the phenomena is extremely challenging for present state-of-the-art theoretical and computational methods, and further development is necessary. Furthermore, the intensity available may already be excessive for the experiments proposed on helium. Perspectives for further development are discussed.

## Introduction

Soon after the invention of optical lasers, harmonic-generation systems were constructed, which allowed the tripling and doubling of the fundamental frequency. These harmonics are generated coherently and so have a fixed phase relationship with respect to the (co-propagating) fundamental. This property was exploited to perform experiments where the phase relationship was of prime importance, i.e., experiments belonging to the two-color, coherent-control class^1,2^. In such experiments, the fundamental typically generates a multiphoton process, while the harmonic gives rise to a single-photon process. Thus the fundamental is usually required to be far more intense than the harmonic to create measurable interference.

The principal motivation for this paper is the need for fresh approaches to implement coherent control experiments in the extreme ultraviolet (XUV) and soft x-ray region. We recently demonstrated such a scheme^3^, but there are many more that could be pursued. Using our previous work as a starting point, we propose additional experiments, and we also analyze to what extent such experiments could be supported by theory. As is often the case in developing fields, the parameters (here the laser intensities and pulse lengths, as well as the target states involved) that are most convenient for experiment are not necessarily the same as those that can be handled by current theoretical methods. In many cases, including the results reported in^3^, the optimal parameters in experiment and theory are widely divergent, and hence it is necessary to pave a path along which they can meet in the near future.

One group of experiments pioneered by Brumer, Shapiro and others^4,5^ is based on the use of the fundamental and its third harmonic. In this approach, typically a resonance is simultaneously excited by the third harmonic in a one-photon process, and by the more intense fundamental via a three-photon process. The two matrix elements for the excitations must have comparable amplitude for this scheme to function, because otherwise the interference is weak. By varying the relative phase of the light pulses, which determines the relative phase of the matrix elements, the outcome of the resonance excitation is controlled. Examples include the product distribution after dissociation^4^, the angular distribution of emitted electrons, the cross section, or other observables such as the line shape of the transition^6–9^. The process is non-linear because the excitation by the first harmonic depends on the third-order susceptibility of the target^10,11^.

A second group of experiments, established by Elliott and co-workers^12,13^, as well as Baranova and co-workers^14–16^, involves interference between phase-locked fundamental and second harmonic radiation. Once again, the fundamental is far more intense than the second harmonic, but there are two differences with respect to the previous case. Firstly, only the angular distribution of the signal varies and not the total cross section^12^. Secondly, the symmetry is broken, and as a result the photoelectron angular distributions are asymmetric. For this situation to occur, it is a necessary (but not a sufficient) condition that the optical response is anharmonic, so again this effect has been classified as non-linear by Franco and Brumer^11^. In addition, they showed that this type of coherent control can be described within a classical model.

Experiments of this kind have until recently been limited to laboratory optical lasers, including those with harmonic generation^17–19^. HHG (High Harmonic Generation) systems, as well as XUV, and x-ray Free Electron Lasers (FELs)^20–24^ constitute new sources of light that enormously expand the range of wavelengths available for laser science. FELs, like pulsed optical lasers, have extremely high peak intensity, high transverse coherence, and ultrashort pulse durations. Pulses from the XUV to the x-ray region with energy up to a few mJ/pulse can be generated, but those based on Self-Amplified Stimulated Emission (SASE) suffer from rather poor longitudinal coherence. FERMI is a seeded light source^24^, and combines the properties of short pulse length and high intensity with a far higher degree of longitudinal coherence than previous FELs based on the SASE process. As well, FERMI is a very versatile machine and can be operated in a number of configurations to control the wavelength, amplitude, polarization and phase of the photons it produces. We have recently shown that the fundamental and second harmonic can be generated coherently^3^, and the relative phase can be controlled with a precision of a few attoseconds. The question immediately arises as to how this capability can be utilized to perform new experiments. This degree of control promises to open up a new field of research: coherent control with x-ray beams, with much shorter wavelengths than previously possible^25^.

The challenges to be met are very great, as many of the techniques used in the optical regime are not applicable for x-rays. A standard method of phase tuning at long wavelengths is to pass the two co-propagating beams through a dispersive gas where the difference in refractive indices for the two wavelengths delays them by different times, and the phase is then adjusted by tuning the pressure. This does not work at wavelengths shorter than the ionisation potential of helium (50.4 nm), as all gases absorb too strongly to provide useful delays. An alternative method is to use mechanical split-and-delay lines. In the soft x-ray region, grazing incidence optics are usually necessary to ensure good reflectivity and acceptable working range, but the mechanical precision of such instruments is usually insufficient to achieve sub-wavelength precision, e.g., one state-of-the-art instrument was reported to have a resolution of 210 attoseconds^26^. Another alternative is to use split-mirror, normal-incidence optics^27,28^, which works well at long wavelength: a resolution of about 40 to 28 attoseconds has been reported for this method. However, for short wavelengths special coatings (such as multilayers) and filters are required to ensure each half of the mirror is illuminated only by the desired harmonic. Generally, filters have narrow working ranges and low efficiencies, so this technique is very limited. Recently a split-and-delay device has been reported with a mechanical resolution of 3 nm corresponding to 7.5 attoseconds^29^. This uses a near normal incidence mirror and so is limited to long wavelengths, or to specially coated mirrors with narrow working ranges.

Recent theoretical work on XUV coherent control of H^30^ and Ne^31,32^ has explored a number of parameters of the experiments, with a view to interpreting the experimental data. The most recent work^32^ discussed in extensive detail the formal derivation of equations describing the observable asymmetry. Here we use these results as a starting point for new calculations, and take a different point of view: we seek to understand to what extent effects are observable under optimal conditions.

This manuscript is organized as follows. The next section describes the method of generating and controlling bichromatic phase-coherent XUV light from a seeded FEL. A very brief description has been given previously^3^, and here we give more detailed information. The following part of the manuscript deals with the theoretical description of possible experiments involving such XUV light, specifically, “first plus second” (*ω* + 2*ω*) and “first plus third” (*ω* + 3*ω*) schemes, both of which are now experimentally possible. The schemes are then illustrated with numerical calculations, and the sensitivity of the predictions to a number of laser parameters as well as the approximations made in various theoretical treatments is discussed. We emphasize that these calculations are not yet able to describe experiments such as the one performed in^3^, due to both the experimental parameters chosen and the remaining large uncertainty in these parameters.

## Generation of Phase-coherent XUV Light From a Seeded FEL

The coherent properties of the light emitted by a free-electron laser are determined by the longitudinal density distribution of an electron beam traveling in a magnetic undulator. The electron density distribution *ρ*(*z*) is periodically modulated at the resonant wavelength *λ* of the undulator, and the radiation field growth rate is proportional to the first coefficient of the discrete Fourier transform of this density distribution,

1 $${b}_{1}(z,\lambda )=\frac{2\pi }{\lambda }{\int }_{z}^{z+\lambda }\rho (z^{\prime} )\,\exp \,(\frac{2\pi iz^{\prime} }{\lambda })dz^{\prime} .$$

The parameter *b*_1_ represents the beam bunching factor calculated at the fundamental resonant frequency of the FEL amplifier at the position *z* along the bunch. Since the FEL is a narrow-bandwidth amplifier centered at *λ*, the bunching factor is a quasi-periodic function of *λ*, only weakly dependent on the coordinate *z*. The density modulation, which evolves in the undulator as a consequence of the interaction of the beam with the combined fields of the undulator and the co-propagating electromagnetic wave, develops substantial Fourier components at higher harmonics of the fundamental when the FEL approaches saturation, and emission at the corresponding wavelengths occurs. There is widespread interest in this process, because emission of high-order harmonics represents a significant resource to extend the operating wavelength range of FELs^33–36^, and also because the simultaneous generation of radiation pulses with multiple frequencies can be implemented in multi-color experiments where the different light beams interact with the sample under study^37^.

Conversely, the generation of harmonics may be undesirable due to interference with multi-photon experiments. Specifically, Nikolopoulos and Lambropoulos^38^ considered the case of neon ionisation in the vicinity of the 2*p*^5^4*s* states by both two-photon resonant and single photon ionisation. They predicted saturation effects to appear at a first harmonic peak intensity of about 10^12^ W/cm^2^ for resonant or near-resonant excitation. The main focus of the paper was on the effect of undesired second harmonic radiation, which is produced in FELs at the level of about 1% or lower of the fundamental intensity. This spurious, incoherent radiation did not cause this kind of problem in our recently reported work^3^, as we will discuss below.

The class of experiments requiring two colors benefits from independent control of the phase relation and amplitude of the radiation emitted at the different wavelengths, and here we present a detailed scheme for doing this. When the electron beam density modulation driven by the interaction with the laser field and the emission at the higher-order harmonics builds up, the field components at the various harmonics are generated by the same current source and therefore have a precise phase correlation with the fundamental. This is of primary importance: the Fourier components are coherent, and this property opens the door to phase control. At saturation, harmonics of the FEL fundamental appear in the emission spectrum. High-order harmonics in high-gain FELs have been measured at different facilities^39–44^, but none of those measurements addressed the phase relation with the FEL fundamental. We have studied the dynamics of this process by solving the Maxwell-Lorentz coupled equations governing the system, with the code PERSEO^45,46^, specifically designed to study the longitudinal dynamics of a FEL.

**Figure 1:**
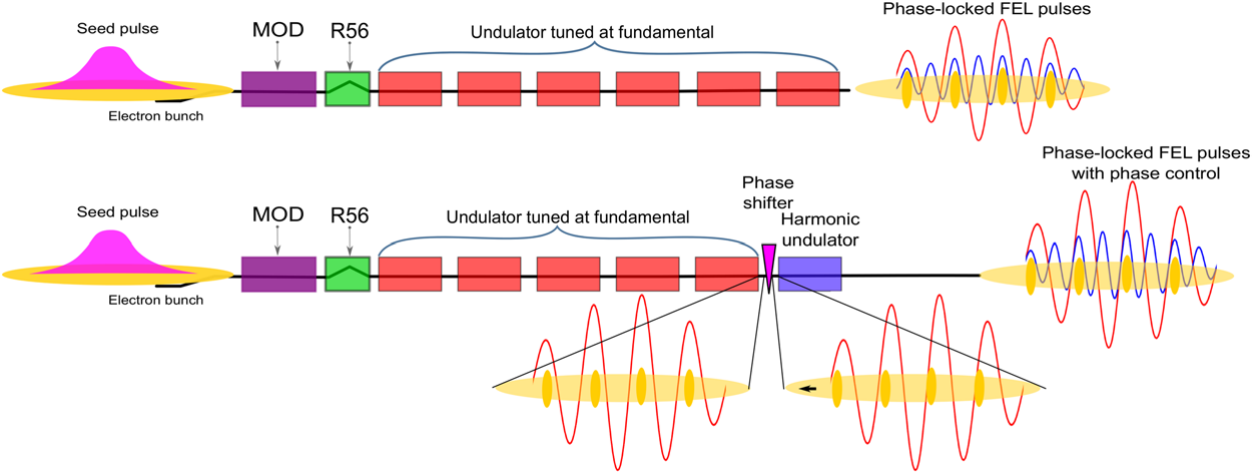
Schematic layout of the FERMI FEL-1 in the two configurations analysed. Upper scheme: fundamental and harmonic radiation are produced by the same undulators using the nonlinear harmonic emission. Lower scheme: the harmonic emission is generated in separate undulators allowing phase control of the two fields.

We have considered the specific layout of the high-gain harmonic generation machine FERMI FEL-1 shown in Fig. 1. In this configuration, the FEL operates as a harmonic converter. A UV pulse is injected into the modulator (MOD) together with the electron beam. The beam is modulated in energy by the simultaneous interaction with the fields of the seed laser and of the modulator itself, and then passes through a dispersive magnetic chicane which converts this energy modulation into a density modulation. The higher-harmonic Fourier component of this modulation, which is resonant with the radiators, is then amplified in the high-gain regime up to saturation. This harmonic of the seed laser is labeled the fundamental wavelength of the FEL.

The intrinsic non-linear harmonic produced during the FEL amplification process, shown in the upper panel of Fig. 1, is the easiest way for generating harmonic emission, but it is limited. While the relative power between the fundamental and the harmonic field can be adjusted by taking advantage of the different growth rates characterizing the two fields, there is very little margin to control the relative phase between the two. Moreover, the method can only be efficiently applied for the third harmonic and for linearly polarized FEL emission, since in the case of even harmonics or for circularly polarized undulators, the harmonic radiation is emitted off-axis^47–51^, and phase coherence is not conserved during beam transport. This is because the off-axis radiation follows a different, and slightly fluctuating, path with respect to the on-axis radiation. In our recent experiment^3^, this undesired radiation was not a significant problem. The ionisation rates for two-photon and single-photon ionisation (by coherent second harmonic light) were adjusted to be approximately equal. The procedure involves attenuating the coherent second harmonic, in that case by a factor of about 10. This has the effect of diminishing the spurious intensity to a level of less than 0.1% of the fundamental radiation.

This intrinsic mechanism operates for SASE light sources, where the pulses typically consist of a series of uncorrelated spikes. Each spike will produce third-harmonic radiation, which is coherent with the fundamental produced by that spike. However, there is a spread in the phase difference for different spikes, as well as variations in the ratio of intensities of the two wavelengths, and a spread in wavelengths over the pulse, up to 1%. This is due to the stochastic nature of SASE, and it is likely that coherent control is impossible with bichromatic SASE light produced in the normal multi-spike mode.

A more suitable solution than the use of intrinsic emission shown in the upper panel of Fig. 1 can be implemented at FERMI by taking advantage of the seeding process that allows accurate control of the startup of the FEL amplification; see lower panel of Fig. 1. By appropriate tuning of the seeding, one can set the FEL so that the high bunching at both the fundamental and at the harmonics is reached before the last undulator and the final undulator can be tuned directly at the desired harmonic. Emission in the last undulator benefits from the bunching present in the electron beam, and coherent harmonic radiation can be generated from a single undulator. Given the fact that the bunching has been generated by the FEL process at the fundamental in the previous undulators, and that the final undulator can only slightly modify it, the emission of the two harmonic fields is expected to be strongly correlated in phase. In this configuration, harmonic emission will mainly be generated in an undulator that is directly tuned on resonance to the desired wavelength and no limitations on polarization or harmonic order exist. Moreover, since the harmonic emission occurs in a separate undulator, the control of the relative phase between fundamental and harmonic radiation is straightforward, utilizing the phase shifters (electron delay lines, already present in the machine) to slightly change the relative phase between the electron beam and the radiation. These devices are extremely simple and consist of small chicanes which lengthen the path of the electrons by very small increments, and are installed between every pair of undulators.

**Figure 2:**
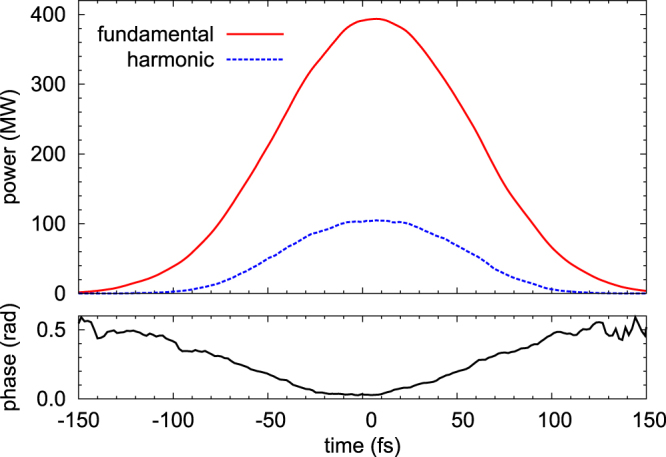
Linearly polarized light, fundamental 62 nm, second harmonic 31 nm. Top curve (red): power of the fundamental; middle curve (dotted blue), second harmonic, as a function of time (*t *= *z*/*c*). Bottom curve (black): relative phase of the fundamental and second harmonic.

This second possibility has been explored with a set of numerical simulations with the software Ginger^52^, and verified by experimental measurements. The Ginger model includes a full three-dimensional description of the generated field (transverse and longitudinal). Figure 2 shows the results of a calculation for the production of first- and second-harmonic FEL radiation, with the parameters shown in Table 1. The results of numerical simulations show that it is possible to set the FEL in a condition where the difference between the phases of the fundamental and higher harmonic pulses is almost constant (and in any case predictable) along the entire FEL pulse, as shown by the black curve in the lower panel of Fig. 2. The realization of this condition strongly depends on the FEL optimization. When the FEL is operated close to saturation in the first set of undulators, significantly higher fluctuations in the relative phase between the fundamental and higher harmonic occur.

**Table 1:** List of FEL parameters for the study of harmonic production.

Electron beam parameters	Value	Units
Mean energy	1.2	GeV
Energy spread	150	keV
Peak current	500	A
Emittance	1.5	mm mrad
Beam size	100	*μ*m
**Laser beam parameters**		
Wavelength	260	nm
Power	40	MW
Pulse length (FWHM)	210	fs

### Theory of experiments with phase-coherent XUV light

In this section, we discuss two experiments to observe the effect of a phase difference in a two-color measurement, similar to Brumer-Shapiro setups in the optical range^5^. Both examples consist of coherent control of the fundamental and its *κ* th harmonic radiation. The electric field is written as

2 $$F(t)=[{F}_{\omega }\,\cos \,(\omega t)+{F}_{\kappa \omega }\,\cos \,(\kappa \omega t-\varphi )]\,{\cos }^{2}\,(\frac{\alpha t}{T}),$$

for $-\,\frac{\pi T}{2\alpha }\le t\le \frac{\pi T}{2\alpha }$, where *ω* is the angular frequency of the fundamental, *ϕ* is the relative phase, *T* is the FWHM of the cosine-squared pulses when $\alpha =\arccos \,\mathrm{(1/}\sqrt[4]{2})\approx 0.572$, while *F*_*ω*_ and *F*_*κω*_ are the amplitudes of the fundamental and the *κ* th harmonic, respectively. Note that *κ* photons of the fundamental frequency are required to get to the same system energy as with one photon of the *κ* th harmonic. For even *κ*, ejected electron waves with opposite parities are produced by the fundamental and the harmonic, while waves with the same parity emerge when *κ* is odd.

In these experiments an atom is irradiated with two coherent, co-propagating beams. If both pulses are linearly polarized along the same direction, the angular distribution of the photoelectrons is described by the expression

3 $$W(\theta )=\frac{{W}_{0}}{4\pi }(1+\sum _{n > 0}{\beta }_{n}{P}_{n}(\cos \,\theta )),$$

where *β*_*n*_ are the anisotropy parameters, *P*_*n*_(*x*) are Legendre polynomials, and *θ* is the angle with respect to the polarization direction.

In lowest (non-vanishing) order perturbation theory, the angle-integrated probability *W*_0_ does not depend on the relative phase *ϕ* between the two harmonics for even *κ*. The odd *β* s, however, vary with *ϕ*, thereby revealing interference between the two ionisation pathways^12,13,30–32^. In contrast, the angle-integrated probability varies with *ϕ* for odd *κ*, while the *ϕ*-dependence of the anisotropy parameters is generally weak. For resonant excitation, the angle-integrated probability does not depend on the phase at all. Hence the most relevant observables to be measured are the anisotropy parameters and the angle-integrated ionisation probability, respectively. We calculated the anisotropy parameters of the first four Legendre polynomials as a function of photon energy, see Supplementary Information. However, a full discussion of these results would involve an excessive amount of data. Supplementary Fig. S1 shows 24 curves, for only two relative phase differences of the fundamental and second harmonic. Such a detailed discussion would obscure the more general ideas which we are exploring. For this reason, we use hereafter the simplified measure of asymmetry described by Eq. (4), below.

**Figure 3:**
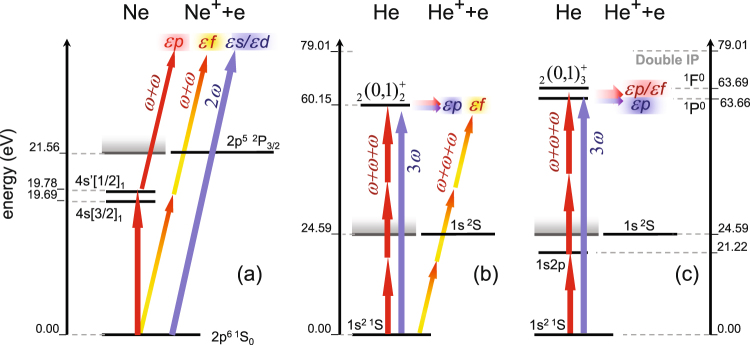
Level scheme for ionisation of neon by the fundamental and its second harmonic (**a**) and helium by the fundamental and its third harmonic (**b**,**c**). The (short) red arrows mark the fundamental photon, while the (long) blue ones indicate the higher harmonic. The horizontal lines show the levels for photoabsorption and autoionisation processes. The relevant energy levels (not to scale) are given on the side of the respective frames.

To perform coherent control with the fundamental plus a second or third harmonic, it is useful to excite a resonance as an intermediate or final state, because a small variation of the photon energy may produce noticeable changes in the ionisation path. Taking into account the typical operation frequencies of FERMI, convenient practical examples are (i) ionisation of Ne by the fundamental and its second harmonic (see Fig. 3a) and (ii) ionisation of He by the fundamental and its third harmonic (see Fig. 3b,c).

Below we will illustrate these schemes by explicit numerical calculations. We will limit the highest peak intensity of the fundamental to 10^13^ W/cm^2^. This is far below the estimated peak intensity of 10^15^ W/cm^2^ used in^3^, but it simplifies the discussion. Increasing the peak intensity by two orders of magnitude, while also using the estimated experimental pulse length, would make the calculations prohibitively expensive. This is an example where both experimental and theoretical efforts will have to be directed towards finding a mutually agreed compromise in the chosen parameters.

### First plus second harmonic

Our first example is the coherent control of the ionisation of neon by the fundamental and its second harmonic radiation. The experimental scheme was recently reported^3^, and a detailed description of the theoretical approaches, comparison with conventional perturbation theory and the role of triplet terms in the continuum can be found in^32^.

As mentioned above, no change in the angle-integrated ionisation probability *W*_0_ occurs when varying the relative phase, because the outgoing waves with different parities are mathematically orthogonal. Consequently, this phenomenon can be observed by monitoring the photoelectron angular distribution, but not by measuring the total ion or electron yields. In particular, the interference between the two ionisation paths produces non-vanishing odd-rank anisotropy *β*-parameters, which in turn cause an asymmetry, defined as

4 $$A{\mathrm{(0}}^{\circ })=\frac{W{\mathrm{(0}}^{\circ })-W{\mathrm{(180}}^{\circ })}{W{\mathrm{(0}}^{\circ })+W{\mathrm{(180}}^{\circ })}=(\sum _{n={\rm{odd}}}{\beta }_{n})/(1+\sum _{n={\rm{even}}}{\beta }_{n})$$

As a specific example, consider resonant photo-excitation of neon via the 2*p*^5^4*s* states: starting from the (2*p*^6^)^1^*S*_0_ ground state with total electronic orbital angular momentum *J* = 0 and even parity, the intermediate resonant state must have *J* = 1 and odd parity. In reality, there are two such states, often labeled 4*s*[3/2]_1_ and 4*s*′[1/2]_1_, which can be excited by 62.974 nm (19.688 eV) and 62.680 nm (19.780 eV) radiation (Fig. 3a). Neither of these states is well *LS*-coupled, but rather a mixture of states with ^1^*P* and ^3^*P* character (with LS-purities of 34% and 66% according to our multiconfiguration Hartree-Fock model^53^). They are well described in an intermediate-coupling framework as a linear combination of *LS*-coupled states. The energy difference is essentially the fine-structure splitting of the Ne(2*p*^5^)^2^*P*_3/2,1/2_ ionic core. Since the ^1^*P* component of these states is the only one that can be excited by a first-order electric dipole transition, one might expect the results to be qualitatively similar for both states. Hence, it should be possible to calculate the asymmetry, at least approximately, in a nonrelativistic framework. Note that three *J* = 1 states with principal configuration 2*p*^5^3*d* are also found a little above the 2*p*^5^4*s* states, see below.

In order to demonstrate the interference effect, angle-resolved spectra were calculated by solving either the single-active-electron nonrelativistic Time-Dependent Schrödinger Equation (TDSE)^54^ or by using lowest-order Perturbation Theory (PT). For the latter, the calculations were performed in both nonrelativistic (PT-LS) and relativistic (PT-J) frameworks. In the nonrelativistic models, only one 2*p*^5^4*s* state with ^1^*P* character exists, which was included with the energy corresponding to the properly averaged 2*p*-4*s* one-electron transition (19.750 eV), while the relativistic (PT-J) framework allows for the proper separation of the 4*s*[3/2]_1_ and 4*s*′[1/2]_1_ states (92 meV). In all calculations (except for those in which the number of cycles was varied), the pulse contained 250 optical cycles with a cosine-squared envelope of the electric field (corresponding to a FWHM of the intensity of ~26.3 fs) and a peak intensity of 10^12^ W/cm^2^ for the first harmonic. The power of the second harmonic was optimized to yield equal contributions to the ionisation probability from the one-photon and two-photon paths when the fundamental is in resonance with the theoretical (model dependent, see above) energies of the respective 2*p*^5^4*s* states. Choosing a ratio of first to second harmonic field amplitudes of 0.02155 (corresponding to an intensity of 0.046 % of the second harmonic relative to the fundamental) provides strong two-pathway interference in this case.

**Figure 4:**
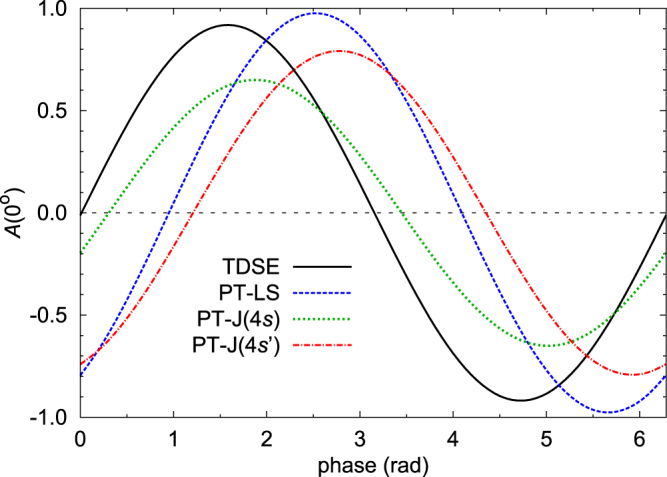
Calculations of the asymmetry *A*(0°) of Ne as a function of the phase between the fundamental frequency and its second harmonic. The central photon energy is fixed at the resonance energy. Predictions from the following models are shown: nonrelativistic TDSE (full black curve); PT-LS (blue dashed curve) at the averaged 2*p*-4*s* resonant transition energy; PT-J, at the *s* (green dotted curve) and *s*’ (red dash-dotted curve) 2*p*^5^4*s J *= 1 states. See text for details.

Figure 4 shows the most important result, namely that the asymmetry strongly depends on the relative phase between the fundamental and the second harmonic. While there are differences in the actual numbers from the various models, the qualitative predictions agree reasonably well. The nonrelativistic models predict values of the asymmetry essentially within the maximum possible interval of [−1, +1], with a sinusoidal dependence on the relative phase. The effect remains in the relativistic PT-J model, even though the amplitude is reduced to about 2/3 of the theoretical maximum. Also, there is a different phase offset between the results for the two *J* = 1 states.

**Figure 5:**
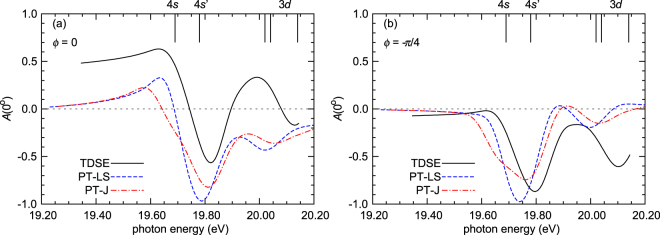
Energy dependence of the asymmetry *A*(0°) for Ne as a function of the relative phase between the harmonics, for *ϕ* = 0 (**a**) and *ϕ* = −*π*/4 (**b**), as predicted by the PT-LS (blue dashed curve), PT-J (red dash-dotted curve) and TDSE (black solid curve) models. The pulse parameters are the same as in Fig. 4. The energies of the fine-structure intermediate states are marked by the vertical lines.

Figure 5 shows what might be expected if the relative phase between the harmonics is kept fixed, but the central photon energy is varied. The TDSE results were shifted so that the resonance energy corresponds to the experimental energy of the 4*s* state. Not surprisingly, the PT-J and PT-LS predictions are similar, except that in the first case, the resonance-like behavior is spread out over the region of intermediate states, and once again, the extrema tend to be reduced. On the other hand, there are significant differences between the PT-LS and TDSE results, particularly for *ϕ* = 0 shown in the panel (a) of Fig. 5.

These remaining disagreements are probably due to a number of differences in the two models. In the TDSE calculations, all single-electron nonrelativistic ^1^*P* states with configurations 2*p*^5^3*s*, 3*d*, 4*s*, 4*d*, 5*s*, 5*d*, … are incorporated, i.e., only one state is included from each configuration. In the second-order PT calculations, on the other hand, we either included three *LS*-coupled ^1^*P* intermediate states with configurations 2*p*^5^3*s*, 2*p*^5^4*s*, and 2*p*^5^3*d* or seven states with these configurations and *J* = 1. These states contained contributions from *LS*-coupled odd-parity ^1^*P*, ^3^*P*, and ^3^*D* states. Furthermore, the TDSE uses a model potential obtained from the density of the occupied one-electron orbitals, while PT employs multi-configuration Hartree-Fock wave functions^53^. Here we only mention that the TDSE calculations predict a strong probability for ejected electrons with orbital angular momentum $\ell =3$, whereas the PT models only show a small effect of such *f*-waves. Since the scattering phase variations for all of the waves are small, the phase difference between the harmonics *ϕ* (actually cos*ϕ*) governs the interference contribution from the *f*-wave. Numerical calculations indicate that for *ϕ* ≈ −*π*/4 this contribution is predicted to be small as a result of the orthogonality of the *f*-amplitude to the combination of *s*- and *d*-amplitudes in the asymmetry expression. (The particular value of *ϕ* depends on the scattering phases of the electron waves.) Indeed, the agreement between TDSE and PT-LS is really good in panel (b) of Fig. 5.

The *f*-wave channel discussed above leads to another very important consequence. The TDSE approach, which accounts for direct two-photon absorption, predicts saturation at an intensity about an order of magnitude higher than that predicted in^38^. In reality, even this estimate is likely to be too low, since the oscillator strength is spread over both fine-structure sublevels. This is expected to nearly double the actual saturation intensity.

A major advantage of PT calculations is their computational efficiency, which allows extensive scans over several parameters that can be controlled in an experiment, such as the relative phase between the harmonics, the photon energy, and the pulse length. The latter, in particular, represents a major challenge in the TDSE formulations for long pulses, since not only the number of cycles increases, but also the radial grid has to be extended in order to account for ejected electrons going far away from the interaction volume while the pulse is still on. On the other hand, PT calculations are limited to relatively low peak intensities. This shows, once again, that even state-of-the-art calculations are by no means straightforward for the experiments under consideration.

**Figure 6:**
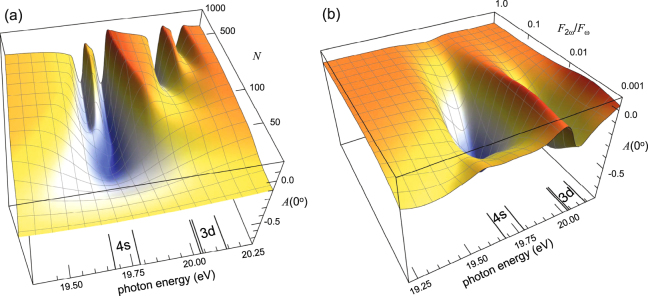
3D plot of the asymmetry *A*(0°) in neon, calculated with the PT-J model for a fundamental peak intensity of 10^12^ W/cm^2^. Predictions as a function of photon energy for the full pulse duration (in optical cycles *N*) and an amplitude ratio *F*_2*ω*_/*F*_*ω*_ = 0.02155 (**a**). The dependence of the results on the ratio of the harmonic amplitudes for *N* = 250 (**b**). In both panels, the relative phase is set to *ϕ* = −*π*/4. The positions of the intermediate states are indicated by lines.

Figure 6a shows the dependence of the asymmetry for a fixed *ϕ* = −*π*/4. Even though the PT-J predictions should only be considered as a qualitative guide, it seems clear that the field of potential investigations is very wide. As expected and clearly seen from Fig. 6a, the effect of the neighboring states depends on the pulse duration. Starting from a long pulse of nearly infinite length (i.e. continuous radiation), the coupling between neighboring states increases with decreasing pulse length, which broadens the resonance structures. In this particular case, however, about 50 cycles, corresponding to a bandwidth of about 0.4 eV, are the minimum required to see a structure in the asymmetry. For shorter pulses, the bandwidth is so large that the resonance structure becomes weaker and eventually invisible. Figure 6b shows the effect of the relative amplitude of the two wavelengths on the asymmetry. Clearly there is an optimum ratio for interference and for large or small values of the ratio, two-photon or single-photon ionisation respectively dominates.

Here we have built on the results of^32^, and considered a different range of parameters in order to optimise the experimental conditions. While that study indicated that a quantitative comparison with published data may be possible, here we reach a different conclusion. We reiterate that a quantitative comparison of the presently possible theoretical predictions with the experimental data of Prince *et al*.^3^ is not meaningful, since the measurements were performed at a single photon energy with a much higher peak intensity than what we can currently handle numerically. We also note that the phase offset varies strongly in the vicinity of a resonance (in classical scattering theory it jumps by *π*), and hence even a small uncertainty in the energy may change the sign of the phase offset. To avoid this problem one should measure at least three energy points, namely at, below, and above the resonance. It is possible, however, to discuss qualitatively the amplitude of the asymmetry. In ref.^3^, the signals from the fundamental and the second harmonic were matched. To do this, the fundamental radiation was used, and the signal due to two-photon and incoherent second harmonic ionization was measured. The signal was then filtered by a gas attenuator transparent to the fundamental, but which absorbed the second harmonic light. The maximum attenuation available with the attenuating gas (helium), was a factor of 10, but it is possible that a small amount of incoherent radiation was present, at the level of 0.1% or less of the fundamental intensity. The coherent second harmonic light was then added and the attenuation adjusted so that the total signal from two-photon and single photon ionization was double the value for two-photon ionization. In any case, the coherent fraction of second harmonic was at least an order of magnitude higher than the incoherent fraction.

### First plus third harmonic

As a second example, we propose a fundamental plus third-harmonic scheme, based on the resonant excitation of helium atoms to doubly excited states of ^1^*P*^*o*^ symmetry. In this experiment, the sample is irradiated with two coherent, co-propagating beams leading via two paths to the same ^1^*P*^*o*^ final state. The interference is again sensitive to the relative phase *ϕ* between the two pulses. The effect can be monitored via the ion yield, as the cross section is predicted to change, via the photoelectron intensity, and via the line shape for autoionisation from resonant states^6,7,17^.

We considered two schemes for this experiment, “singly resonant” and “doubly resonant”, as shown in Fig. 3(b,c). The experimental energies of the resonant states have been reported^55^ and we consider the two states labeled in that work (2, 0)_2_ or 2*s*2*p* ^1^*P*^*o*^, and (2, 0)_3_ or 2*s*3*p* ^1^*P*^*o*^. Here we use the notation of Lin^56^, ${}_{N}{}^{}(K,T)_{n}^{A}\,{}_{}{}^{2S+1}L_{}^{{\rm{\prod }}}={}_{2}{}^{}(0,1)_{2}^{+}\,{}_{}{}^{1}P_{}^{o}$ and ${}_{2}{}^{}(0,1)_{3}^{+}\,{}_{}{}^{1}P_{}^{o}$ respectively.

We consider first the singly resonant scheme: the fundamental photon energy of 20.05 eV is chosen to be resonant with the doubly excited state at 60.15 eV relative to the ground state^55^ via a three-photon process, while the third harmonic excites this state directly. The doubly excited ${}_{2}{}^{}(0,1)_{2}^{+}\,{}_{}{}^{1}P_{}^{o}$ state decays via autoionisation to a helium ion in the ground state and an ejected electron with energy 35.56 eV. The third harmonic can excite only ^1^*P*^*o*^ states, as it is a single photon process, whereas the fundamental can excite both ^1^*P*^*o*^ and ^1^*F*^*o*^ states via a three-photon process. Lipsky *et al*.^57^ calculated that the first ^1^*F*^*o*^ resonance lies at 63.69 eV, far from the present energy, so the ionisation to the ^1^*F*^*o*^ states with the fundamental is expected to be very weak as it is a non-resonant three-photon process. Therefore, interference can only occur in the ^1^*P*^*o*^ states in this scheme. If the photon energy is not chosen to be in the vicinity of a doubly excited ^1^*P*^*o*^ resonance, the additional ^1^*F*^*o*^ states have to be taken into account as well.

Calculations were performed by solving the TDSE with the methods and approach described in^58,59^. In addition, to demonstrate the interference effects, we identified the FEL parameter space, such as intensities and pulse durations, needed to observe experimentally coherent control of the photoionisation yield. The electric field of the laser pulses of the fundamental and the third harmonic is described according to Eq. (2), where now *F*_3*ω*_ is the amplitude of the third harmonic.

**Figure 7:**
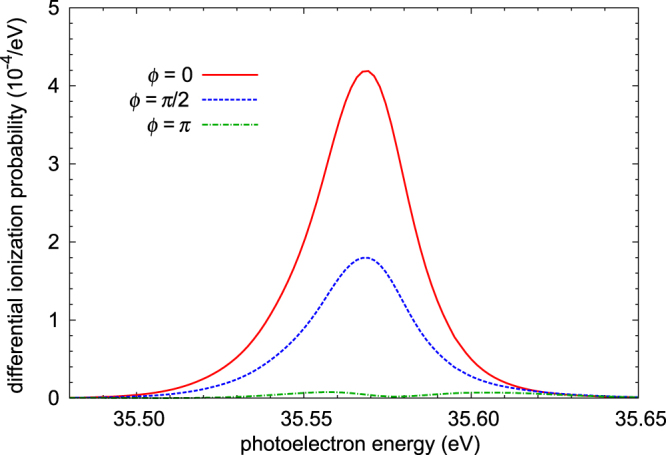
Calculated photoelectron spectra of He for fundamental wavelength irradiance of 10^13^W/cm^2^, third-harmonic irradiance of 2 × 10^8^ W/cm^2^, pulse duration of 33.2 fs, and relative phase *ϕ* between the fundamental and third harmonic of 0, *π*/2 or *π* rad. Photon energies: fundamental, 20.05 eV; third harmonic, 60.15 eV.

Figure 7 shows the results of two-color calculations of photoelectron energy spectra as an example for the singly resonant case. In this example, the intensities of the fundamental and the third harmonic are 10^13^ W/cm^2^, and 2 × 10^8^ W/cm^2^, respectively. The two pulses have the same duration of 33.2 fs. Destructive interference is visible when the third harmonic and fundamental are out of phase (*ϕ* = *π*), in which case the emission is almost completely suppressed. This result demonstrates that, as expected, control of the double electron excitation can be achieved. Complete cancellation of the photoelectron spectrum is unlikely, since the line shapes with single- and three-photon processes are different.

Now we relate the calculations to the experimental conditions available at FERMI. Our calculations are restricted to pulse durations less than 40 fs due to the limits of our computational capacity. In order to provide laser parameters needed to observe experimentally coherent control of the photoionisation yield, we have to extrapolate the current results to the typical pulse duration of 100 fs at FERMI to find comparable single-color transition amplitudes for the fundamental and the third harmonic, as long as the laser parameters are within the perturbative regime. For example, for a given pulse duration, the three-photon absorption probability should scale as the cubic power of the laser intensity, as predicted by third-order perturbation theory, while the one-photon absorption probability scales linearly. Deviation from this power law dependence indicates a breakdown of the perturbation theory of the corresponding order.

For a fundamental pulse of duration less than 40 fs, our calculations show that the ionisation probability precisely follows the cubic power law for intensities less than 10 TW/cm^2^, while third-order perturbation theory certainly breaks down for intensities higher than 50 TW/cm^2^. The dependence of the photoionisation probability on the pulse duration can be estimated by perturbation theory for both the one- and three-photon absorption processes as shown in^58,60^. The dependence for one-photon absorption by first-order perturbation theory can even be derived analytically, using the known Fano resonance shape parameters and the width for the ${}_{2}{}^{}(0,1)_{2}^{+}\,{}_{}{}^{1}P_{}^{o}$ state. The three-photon process can also be modeled by a two-step model based on perturbation theory, where the helium atom is excited by one-photon absorption to the 1*s*2*p* ^1^*P*^*o*^ state, followed by two-photon absorption to the ${}_{2}{}^{}(0,1)_{2}^{+}\,{}_{}{}^{1}P_{}^{o}$ state. Here the 1*s*2*p* ^1^*P*^*o*^ state is considered as the major intermediate state in the perturbation theory, although the state is not exactly matched to the resonance condition; see^58^ for a detailed description of this approximation. We approximate the density of states near the final ${}_{2}{}^{}(0,1)_{2}^{+}\,{}_{}{}^{1}P_{}^{o}$ state by a Lorentzian form in the energy integration in the perturbative analysis. The result is not sensitive to the choice of the form of the density of states for pulse durations that are long compared to the autoionisation lifetime of about 17 fs.

Assuming an FEL pulse duration of 100 fs, the intensity of the fundamental is fixed at 10^13^ W/cm^2^ to stay within the perturbative regime. To achieve the same transition amplitude as the three-photon absorption of the fundamental, the third harmonic is estimated to have an intensity ~3 × 10^8^ W/cm^2^. The Low Density Matter beamline at FERMI^61^ can presently reach irradiance above 10^14^ W/cm^2^, so the estimated setting of the fundamental can be achieved. Experimentally, setting the ratio of the first to third harmonic intensity to the suggested value of 3 × 10^4^ is a challenge, but may be feasible. If the fundamental intensity can be increased, staying within the perturbative regime, the cubic dependence of the ionisation probability on the intensity could reduce the estimated ratio. However, the improvement is limited.

**Figure 8:**
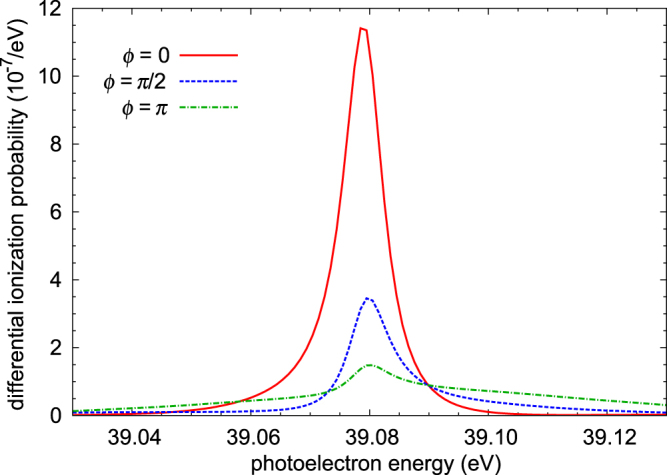
Calculated photoelectron spectrum for first-harmonic intensity of 5 × 10^11^ W/cm^2^, third-harmonic intensity of 10^6^ W/cm^2^, pulse duration of 33.2 fs, and relative phase *ϕ* between the fundamental and third harmonic of 0, *π*/2 or *π* rad. Photon energies: fundamental, 21.22 eV; third harmonic, 63.65 eV.

Next we consider the doubly resonant scheme, depicted in Fig. 3c, where the 1*s*2*p* ^1^*P*^*o*^ state at 21.218 eV^62^ is excited resonantly by the first harmonic. With further two-photon absorption, the doubly excited ${}_{2}{}^{}(0,1)_{3}^{+}\,{}_{}{}^{1}P_{}^{o}$ (or 2*s*3*p* + ^1^*P*^*o*^) state at 63.658 eV is reached^55^. Within the lifetime width of the upper state (10 meV), the energy is exactly three times the excitation energy of the 1*s*2*p* ^1^*P*^*o*^ state^55^. Thus the doubly excited ${{}_{2}(0,1)}_{3}^{+}$ state can be excited by a three-photon (resonant, non-resonant continuum, resonant) process, as well as by a single-photon direct process with the third harmonic. Figure 8 shows an example of how the photoelectron spectrum changes as the relative phase between the fundamental and the third harmonic varies. In this example, the intensities of the fundamental and the third harmonic are 5 × 10^11^ W/cm^2^ and 10^6^ W/cm^2^, respectively. Both pulses have the same duration of 33.2 fs. The intensity chosen for the fundamental in this calculation is easily achievable at FERMI. However, the third harmonic intensity required is very low, which presents experimental challenges. In addition, even at this low intensity and moderate pulse duration of the fundamental, the ground state is predicted to be depleted by 50% due to the onset of Rabi oscillations. For a realistic pulse duration of the order of 100 fs at FERMI, therefore, one has to use a lower intensity to stay within the perturbative regime. As a result, it is very difficult to achieve comparable transition amplitudes between the one- and three-photon processes, since an even weaker third harmonic is needed.

As in the previous singly resonant scheme, the three-photon path may excite both ^1^*P*^*o*^ and ^1^*F*^*o*^ states. The ^1^*F*^*o*^ resonance energy, 63.69 eV, is within the bandwidth of the photons tuned to the excitation to the 1*s*2*p* ^1^*P*^*o*^ state, provided the pulse duration is short enough, so this ^1^*F*^*o*^ resonance can in principle be excited. However, we do not see substantial evidence for this resonance in our calculations. If there is a three-photon ^1^*F*^*o*^ resonance overlapping a ^1^*P*^*o*^ resonance within the bandwidth, as in double-electron excitation in the *N* = 3 manifold^58^, the interference can, in principle, be observed in the photoelectron angular distribution. However in the present case, we estimate the ^1^*F*^*o*^ contribution to be <2%, which is negligible.

We also note that the resonance shapes are sensitive to the relative phase for both the singly and doubly resonant schemes, as seen in Figs 7 and 8. The Fano resonance shape parameter is intimately connected with the phase and the time evolution of resonances^63^, and the present method allows control of the resonance shape parameters as in the case of optical excitation^7^.

## Conclusions and Outlook

We have presented a detailed description of how a seeded Free-Electron Laser can be used to perform some coherent-control experiments using the fundamental and second and third harmonics. The flexible design of FERMI provides excellent control of the wavelength, phase, and amplitude of light emitted by the FEL. Two kinds of experiment have been proposed: first plus second harmonic, for which we have previously published initial experimental results, and first plus third harmonic, which we intend to pursue in the future. This technique opens the way to coherent control with short-wavelength multicolor experiments, in which the amplitude, wavelength, and phase can be controlled. The calculations for experiments with the third harmonic provide a surprising result: the intensity available may already be too great to observe the effects we seek. In the non-resonant case, normally available intensities may already be out of the perturbative regime, thereby complicating the process considerably. In the double-resonant case, a typical intensity will probably deplete the sample strongly or at least induce Rabi oscillations, thereby again complicating and possibly masking the desired effects.

We hope that the ideas and model calculations presented in this paper will encourage both experimentalists and theorists to push current capabilities to a parameter regime that makes experiments possible while also being treatable by *ab initio* numerical methods. A prime requirement is much higher computing power, which will gradually become available as computers become more powerful. For wavelengths near a resonance with strong configuration mixing (for example 4*s* of neon), a multi-electron theory would be needed that accounts at least approximately for the relativistic intermediate-coupling effects in the excited states. As illustrated for the case of helium, state-of-the-art calculations are prohibitively expensive in terms of computing time for the current pulse length available at FERMI. This may be remedied by more powerful computers, or by accelerator physics developments leading to pulses of shorter duration. For reliable comparison of experiment with theory, it is crucial to measure with different wavelengths, including off resonance. Besides, a target with more isolated resonances would provide clearer results. Finally, experience with optical lasers has taught that pioneering work often begins with atomic and molecular targets to establish feasibility and methods, which are later applied to condensed matter. Thus we foresee that the methods developed here at short wavelengths will in the future be applied to solid-state samples to control the outcome of light-matter interactions.

## Electronic supplementary material

Supplementary information

## References

[1] Brif C, Chakrabarti R, Rabitz H (2010). Control of quantum phenomena: past, present and future. New J. Phys..

[2] Ehlotzky F (2001). Atomic phenomena in bichromatic laser fields. Phys. Rep..

[3] Prince KC (2016). Coherent control with a short-wavelength Free Electron Laser. Nat. Photonics.

[4] Chan CK, Brumer P, Shapiro M (1991). Coherent radiative control of IBr photodissociation via simultaneous (1,3) excitation. J. Chem. Phys..

[5] Brumer, P. & Shapiro, M. Principles of the Quantum Control of Molecular Processes (Wiley-VCH, 2003).

[6] Nakajima T, Lambropoulos P, Cavalieri S, Matera M (1992). Modulating Ionization through Phase Control. Phys. Rev. A.

[7] Nakajima T, Lambropoulos P (1993). Manipulation of the Line Shape and Final Products of Autoionization through the Phase of the Electric Fields. Phys. Rev. Lett..

[8] Nakajima T, Lambropoulos P (1994). Effects of the Phase of the Laser Field on Autoionization. Phys. Rev. A.

[9] Lambropoulos P, Nakajima T (1999). Origin of the phase lag in the modulation of photoabsorption products under two-color fields. Phys. Rev. Lett..

[10] Franco I, Brumer P (2006). Laser-Induced Spatial Symmetry Breaking in Quantum and Classical Mechanics. Phys. Rev. Lett..

[11] Franco I, Brumer P (2008). Minimum requirements for laser-induced symmetry breaking in quantum and classical mechanics. J. Phys. B: At. Mol. Opt. Phys..

[12] Chen C, Yin Y-Y, Elliott DS (1990). Interference between Optical Transitions. Phys. Rev. Lett..

[13] Yin Y-Y, Chen C, Elliott DS, Smith AV (1992). Asymmetric Photoelectron Angular Distributions from Interfering Photoionization Processes. Phys. Rev. Lett..

[14] Baranova NB, Zel’dovich B, Ya., Chudinov AN, Shul’ginov AA (1990). Theory and observation of polar asymmetry of photoionization in a field with 〈E^3^〉 ≠ 0. Sov. Phys. JETP.

[15] Baranova NB, Zel’dovich BYa (1991). Physical effects in optical fields with nonzero average cube, 〈E^3^〉. J. Opt. Soc. Am. B.

[16] Baranova NB (1992). Observation of an interference of one- and two-photon ionization of the sodium 4s state. JETP Lett..

[17] Nakajima T, Zhang J, Lambropoulos P (1997). Controlling the Branching Ratio of Photoionization Products under Two-Color Excitation: Competition between ac Stark Splitting and Two-Path Interference. Phys. Rev. Lett..

[18] Schumacher DW, Bucksbaum PH (1996). Phase dependence of intense-field ionization. Phys. Rev. A.

[19] Zhu L (1995). Coherent laser control of the product distribution obtained in the photoexcitation of HI. Science.

[20] Ullrich J, Rudenko A, Moshammer R (2012). Free-Electron Lasers: New Avenues in Molecular Physics and Photochemistry. Annu. Rev. Phys. Chem..

[21] Tiedtke K (2009). The soft x-ray free-electron laser FLASH at DESY: beamlines, diagnostics and end-stations. New J. Phys..

[22] Emma P (2010). First lasing and operation of an ångstrom-wavelength free-electron laser. Nat. Photonics.

[23] Ishikawa T (2012). A compact X-ray free-electron laser emitting in the sub-ångström region. Nat. Photonics.

[24] Allaria E (2012). Highly coherent and stable pulses from the FERMI seeded free-electron laser in the extreme ultraviolet. Nat. Photonics.

[25] Hartmann N, Glownia JM (2016). Attosecond coherent control at FELs. Nat. Photonics.

[26] Sorgenfrei F (2010). The extreme ultraviolet split and femtosecond delay unit at the plane grating monochromator beamline PG2 at FLASH. Rev. Sci. Instrum..

[27] Tzallas P, Charalambidis D, Papadogiannis NA, Witte K, Tsakiris GD (2003). Direct observation of attosecond light bunching. Nature.

[28] Takahashi EJ, Lan P, Mucke OD, Nabekawa Y, Midorikawa K (2013). Attosecond nonlinear optics using gigawatt-scale isolated attosecond pulses. Nat. Commun..

[29] Usenko S (2017). Attosecond interferometry with self-amplified spontaneous emission of a free-electron laser. Nat. Commun..

[30] Grum-Grzhimailo AN, Gryzlova EV, Staroselskaya EI, Venzke J, Bartschat K (2015). Interfering onephoton and two-photon ionization by femtosecond VUV pulses in the region of an intermediate resonance. Phys. Rev. A.

[31] Douguet N, Gryzlova EV, Staroselskaya EI, Bartschat K, Grum-Grzhimailo AN (2017). Photoelectron angular distribution in two-pathway ionization of neon with femtosecond XUV pulses. Eur. Phys. J. D.

[32] Gryzlova EV, Grum-Grzhimailo AN, Staroselskaya EI, Douguet N, Bartschat K (2018). Quantum coherent control of the photoelectron angular distribution in bichromatic-field ionization of atomic neon. Phys. Rev. A.

[33] Colson WB (1981). The nonlinear wave equation for higher harmonics in free-electron lasers. IEEE J. Quantum Electron..

[34] Baccaro S, De Martini F, Ghigo A (1982). Charge density harmonics generation in free-electron relativistic parametric devices. Opt. Lett..

[35] Freund HP, Biedron SG, Milton SV (2000). Nonlinear harmonic generation in free-electron lasers. IEEE J. Quantum Electron..

[36] Biedron SG (2002). Exotic harmonic generation schemes in high-gain, free-electron lasers. Proc. SPIE.

[37] Bencivenga F, Masciovecchio C (2009). FEL-based transient grating spectroscopy to investigate nanoscale dynamics. Nucl. Instrum. Methods Phys. Res. A.

[38] Nikolopoulos GM, Lambropoulos P (2015). Resonantly enhanced multiphoton ionization under XUV FEL radiation: a case study of the role of harmonics. J. Phys. B: At. Mol. Opt. Phys..

[39] Biedron SG (2002). Measurements of nonlinear harmonic generation at the Advanced Photon Source’s SASE FEL. Nucl. Instrum. Methods Phys. Res. A.

[40] Ackermann W (2007). Operation of a free-electron laser from the extreme ultraviolet to the water window. Nat. Photonics.

[41] Giannessi L (2012). High-Order-Harmonic Generation and Superradiance in a Seeded Free-Electron Laser. Phys. Rev. Lett..

[42] Ratner D (2011). Second and third harmonic measurements at the linac coherent light source. Phys. Rev. ST Accel. Beams.

[43] Giannessi, L. *et al*. First lasing of FERMI FEL-2 (1st Stage) and FERMI FEL-1 recent results. *Proc. FEL2012, Nara, Japan MOOB6*, https://accelconf.web.cern.ch/accelconf/FEL2012/papers/moob06.pdf (2012).

[44] Allaria E (2008). Experimental characterization of nonlinear harmonic generation in planar and helical undulators. Phys. Rev. Lett..

[45] Giannessi L (2003). Simulation codes for high brightness electron beam free-electron laser experiments. Phys. Rev. ST Accel. Beams.

[46] Giannessi, L. Overview of Perseo, a system for simulating FEL dynamics in Mathcad. *Proc. FEL 2006, BESSY, Berlin, Germany, 91*, https://accelconf.web.cern.ch/accelconf/f06/papers/mopph026.pdf (2006).

[47] Kincaid BM (1977). A short-period helical wiggler as an improved source of synchrotron radiation. J. Appl. Phys..

[48] Barbini R, Ciocci F, Dattoli G, Giannessi L (1990). Spectral properties of the undulator magnets radiation: Analytical and numerical treatment. Riv. Nuovo Cimento.

[49] Saldin EL, Schneidmiller EA, Yurkov MV (2006). Properties of the third harmonic of the radiation from selfamplified spontaneous emission free electron laser. Phys. Rev. ST Accel. Beams.

[50] Geloni G, Saldin E, Schneidmiller E, Yurkov M (2007). Exact solution for second harmonic generation in XFELs. Opt. Commun..

[51] Allaria E, Ninno GD, Geloni G, Spezzani C (2011). Angular distribution of nonlinear harmonic generation in helical undulators: A comparison between experiments and theory. Nucl. Instrum. Methods Phys. Res. A.

[52] Fawley, W. A User Manual for GINGER and its Post-Processor XPLOTGIN, Lawrence Berkeley National Laboratory, Report LBNL-49625 (2004).

[53] Froese Fischer C, Brage T, Jönsson P (1997). Computational Atomic Structure. An MCHF Approach.

[54] Grum-Grzhimailo AN, Kondorskiy AD, Bartschat K (2004). Controlling the angular distribution of atomic photoelectrons in the region of laser-induced continuum structure in the femtosecond time domain. J. Phys. B: At. Mol. Opt. Phys.

[55] Domke M, Schulz K, Remmers G, Kaindl G, Wintgen D (1996). High-resolution study of P doubleexcitation states in helium. Phys. Rev. A.

[56] Lin CD (1986). Doubly excited states, including new classification schemes. Adv. At. Mol. Phys..

[57] Lipsky L, Anania R, Conneely MJ (1977). Energy levels and classifications of doubly-excited states in twoelectron systems with nuclear charge Z = 1, 2, 3, 4, 5, below the N = 2 and N = 3 thresholds. At. Data Nucl. Data Tables.

[58] Liu C-N, Hishikawa A, Morishita T (2012). Two-electron dynamics in nonlinear double excitation of helium by intense ultrashort extreme-ultraviolet pulses. Phys. Rev. A.

[59] Morishita T (2001). Two-electron dynamics in nonlinear double excitation of helium by intense ultrashort extreme-ultraviolet pulses. J. Phys. B: At. Mol. Opt. Phys..

[60] Hishikawa A (2011). Enhanced nonlinear double excitation of He in intense extreme ultraviolet laser fields. Phys. Rev. Lett..

[61] Lyamayev V (2013). A modular end-station for atomic, molecular, and cluster science at the low density matter beamline of FERMI@Elettra. J. Phys. B: At. Mol. Opt. Phys.

[62] Kramida, A., Ralchenko, Yu., Reader, J. & NIST ASD Team. NIST Atomic Spectra Database (ver. 5.4), [online]. Available: http://physics.nist.gov/asd [2017, March 11]. National Institute of Standards and Technology, Gaithersburg, MD (2016).

[63] Ott C (2013). Lorentz Meets Fano in Spectral Line Shapes: A Universal Phase and Its Laser Control. Science.

