# Supplementary material for: Coherent control schemes for the photoionization of neon and helium in the Extreme Ultraviolet spectral region
Source: Sci Rep. 2018 May 17;8:7774. doi: 10.1038/s41598-018-25833-7 (PMC5958097; doi:10.1038/s41598-018-25833-7)
Supplement: Supplementary file 1 — Supplementary information [file 41598_2018_25833_MOESM1_ESM.pdf]

Coherent control schemes for the photoionization of neon and helium in the Extreme Ultraviolet spectral region

Luca Giannessi[1,2], Enrico Allaria[1], Kevin C. Prince[1,*], Carlo Callegari[1], Giuseppe Sansone[3,4], Kiyoshi Ueda[5], Toru Morishita[6], Chien Nan Liu[7], Alexei N. Grum-Grzhimailo[8], Elena V. Gryzlova[8], Nicolas Douguet[9,10], and Klaus Bartschat[9]

[1]Elettra-Sincrotrone Trieste, 34149 Basovizza, Trieste, Italy
[2]ENEA C.R. Frascati, 00044 Frascati, Italy
[3]Dipartimento di Fisica, CNR-IFN, Politecnico di Milano, 20133 Milan, Italy
[4]Physikalisches Institut der Albert-Ludwigs-Universit ¨ at Freiburg, 79104 Freiburg, Germany
[5]Institute of Multidisciplinary Research for Advanced Materials, Tohoku University, Sendai 980-8577, Japan
[6]Institute for Advanced Science, The University of Electro-communications, 1-5-1 Chofu-ga-oka, Chofu-shi, Tokyo 182-8585, Japan
[7]Department of Physics, Fu-Jen Catholic University, Taipei 24205, Taiwan
[8]Skobeltsyn Institute of Nuclear Physics, Lomonosov Moscow State University, Moscow 119991, Russia
[9]Department of Physics and Astronomy, Drake University, Des Moines, Iowa 50311, USA
[10]Department of Physics, University of Central Florida, Orlando, Florida 32816, USA
[*]Kevin.Prince@Elettra.Eu

Supplementary information.

Calculated values of the anisotropy parameters of the Legendre polynomials.

Supplementary Figure S1. Calculated values of the β_n (n=1,..,4) anisotropy parameters of the Legendre polynomials P_n, for the calculational methods TDSE, PT-LS and PT-J, as a function of photon energy (eV), and for different phase differences ϕ between the first and second harmonics. (a) TDSE, ϕ=0. (b) TDSE ϕ=-π/4. (c) PT-LS, ϕ=0. (d) PT-LS, ϕ=-π/4. (e) PT-J, ϕ=0. (f) PT-J, ϕ=-π/4.

The β_2 parameters have a generally similar overall form, with the contrast and absolute values varying a little between different calculational methods. Because even anisotropy parameters are the result of the incoherent sum of single and two-photon processes, β_2 approaches the conventional value for Ne ionization below the resonance, and approaches a value of 2 at the resonance; for a weaker second harmonic, and a longer pulse, the value is closer to 2. Neither β_2 nor β_4 depend on the phase offset ϕ. Similarly the β_1 and β_3 parameters, which determine the observed asymmetry, have similar overall shapes. The spectral features near the 4*s* and 4*s*` resonances are similar for the three theoretical methods, but the TDSE calculations shows stronger modulation near the 3*d* resonances due to the fact that the transition probability which was spread over three 3d fine structure states for the model potential is concentrated into one state.

As noted in the section “First plus second harmonic” of the main text, the TDSE calculations predict a strong probability for ejected electrons with orbital angular momentum *l* = 3, whereas the PT models only

show a small effect of such *f*-waves. This is reflected in the anisotropy parameters, as for the two PT schemes, β_4 does not vary strongly. However, for the TDSE approach, a substantial modulation as a function of photon energy is predicted.

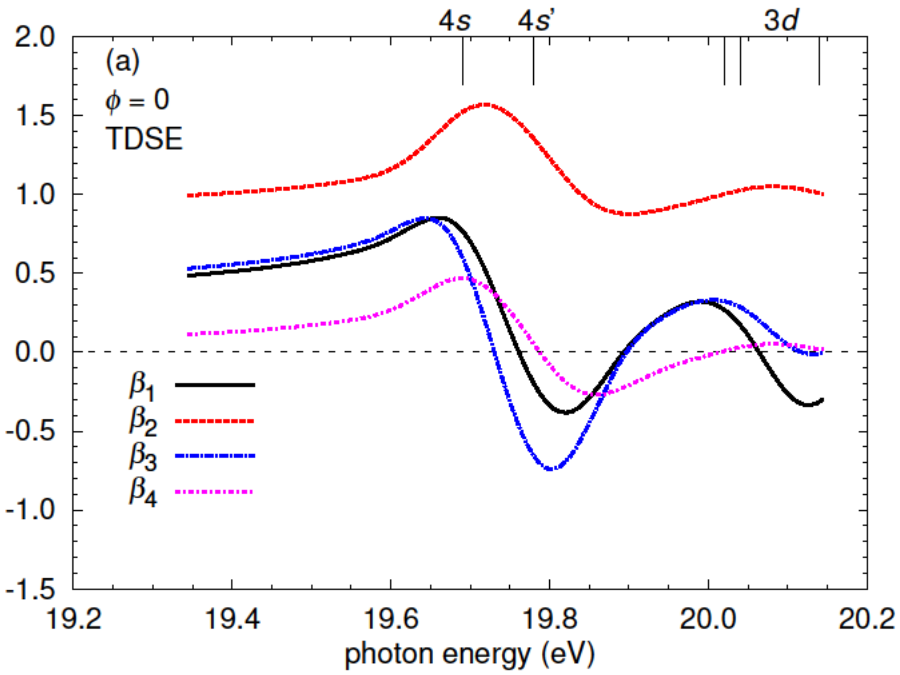

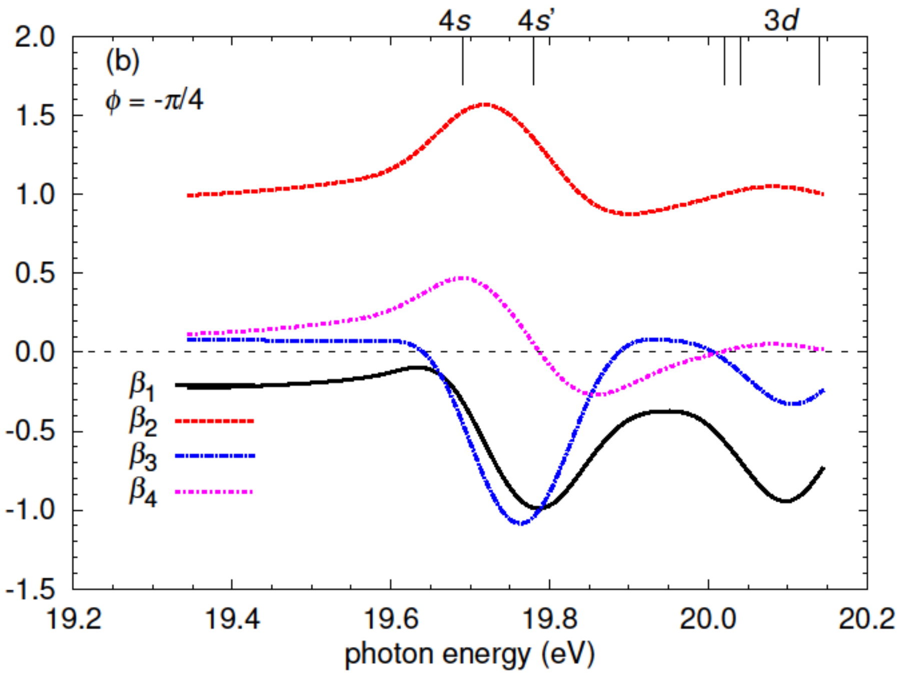

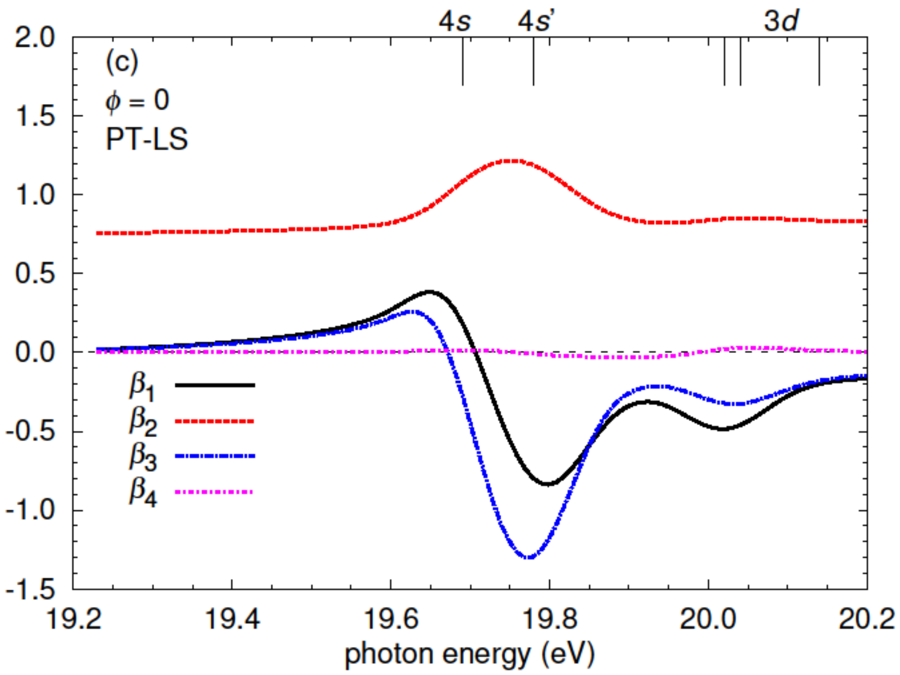

4s
4s'
3d
(c)
ϕ = 0
PT-LS
β_1
β_2
β_3
β_4
2.0
1.5
1.0
0.5
0.0
-0.5
-1.0
-1.5
19.2
19.4
19.6
19.8
20.0
20.2
photon energy (eV)

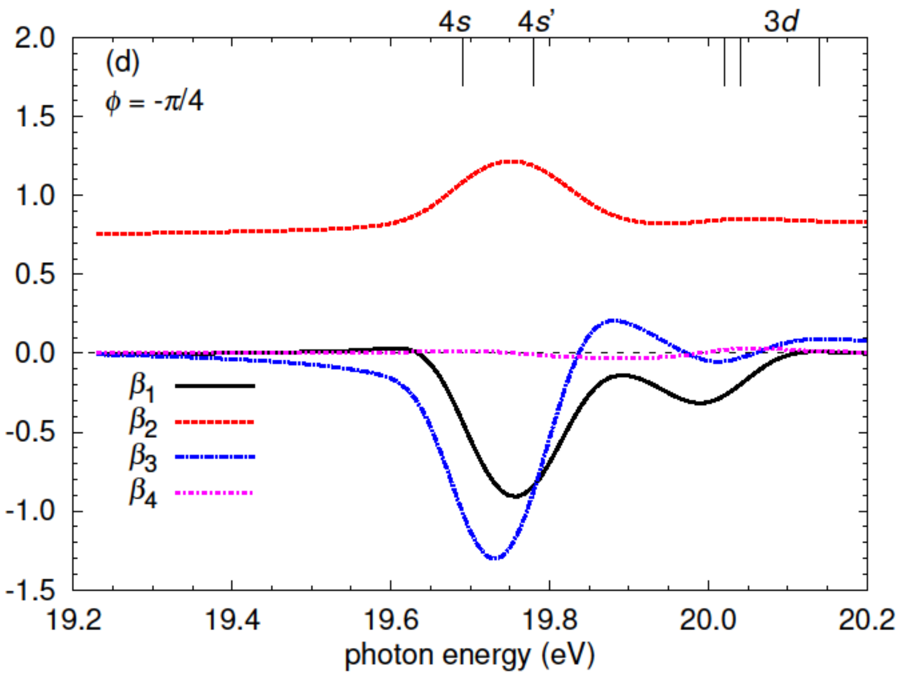

4s
4s'
3d
(d)
ϕ = -π/4
β_1
β_2
β_3
β_4
2.0
1.5
1.0
0.5
0.0
-0.5
-1.0
-1.5
19.2
19.4
19.6
19.8
20.0
20.2
photon energy (eV)

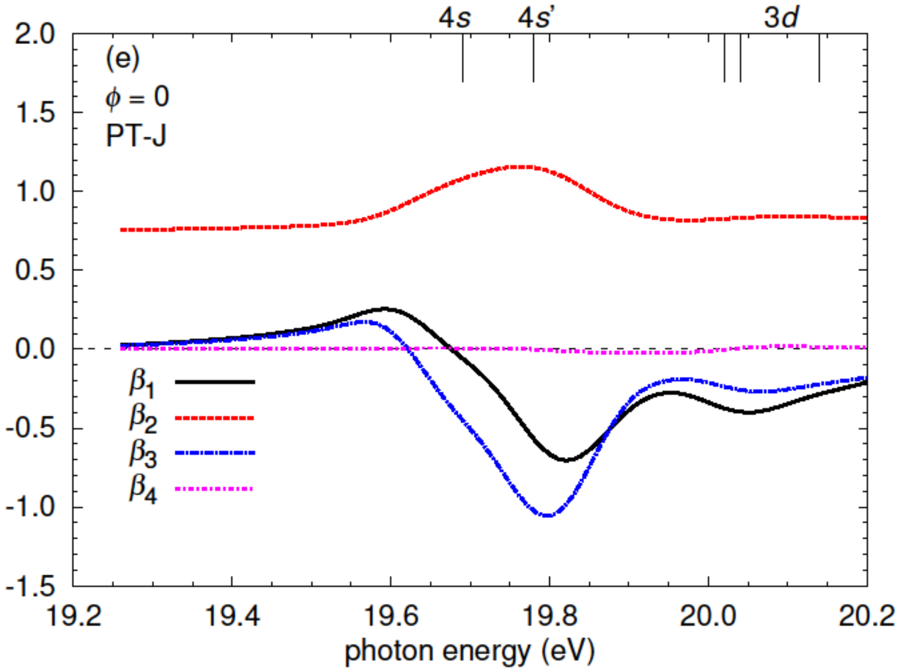

4s
4s'
3d
(e)
ϕ = 0
PT-J
β_1
β_2
β_3
β_4
2.0
1.5
1.0
0.5
0.0
-0.5
-1.0
-1.5
19.2
19.4
19.6
19.8
20.0
20.2
photon energy (eV)

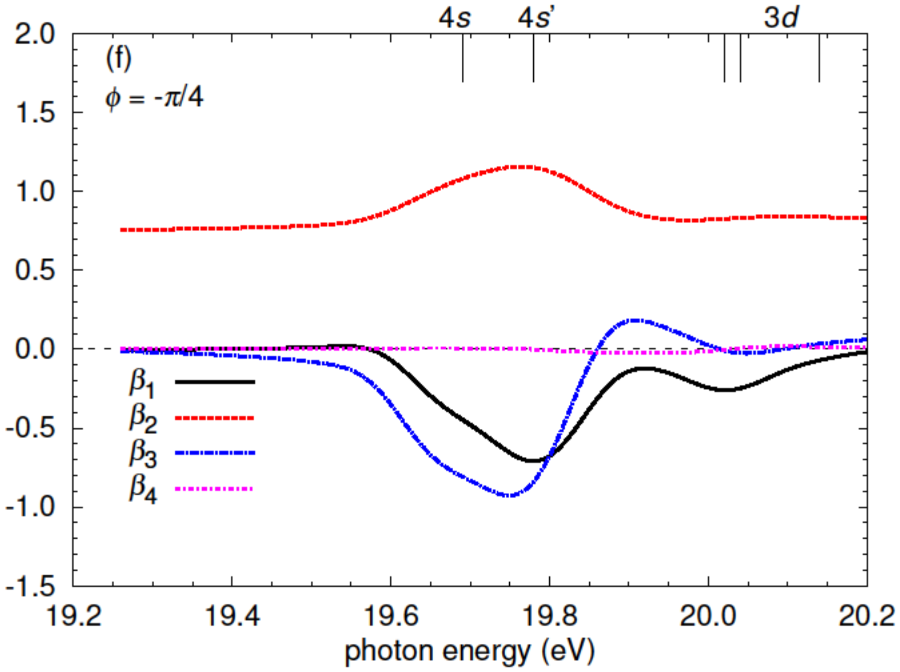

4s
4s'
3d
(f)
ϕ = -π/4
β_1
β_2
β_3
β_4
2.0
1.5
1.0
0.5
0.0
-0.5
-1.0
-1.5
19.2
19.4
19.6
19.8
20.0
20.2
photon energy (eV)
